# Exploring User Experiences With the Lift App for Emotional Well-Being Among Youth With Type 1 Diabetes: Qualitative Study

**DOI:** 10.2196/79896

**Published:** 2026-05-28

**Authors:** Katie M Babbott, Kristin Harrison Ginsberg, Anna L Boggiss, Joanna McClintock, Chloe Hudson, Paul Hofman, Ryan Paul, Alana Cavadino, Hiran Thabrew, Craig Jefferies, Martin de Bock, Jennifer L Sherr, Anna Serlachius

**Affiliations:** 1 Department of Psychological Medicine, Faculty of Medical and Health Sciences, University of Auckland, 85 Park Road, Grafton, Auckland, 1023, New Zealand, 64 93737599 ext 83073; 2 Rio Tinto Children's Diabetes Centre, The Kids Research Institute Australia, Perth Children's Hospital, Perth, Australia; 3 Waikato District, Te Whatu Ora Health New Zealand, Hamilton, New Zealand; 4 Canterbury District, Te Whatu Ora Health New Zealand, Christchurch, New Zealand; 5 Liggins Institute, University of Auckland, Auckland, New Zealand; 6 School of Health Equity and Innovation, Division of Health, University of Waikato, Hamilton, New Zealand; 7 Epidemiology and Biostatistics, University of Auckland, Auckland, New Zealand; 8 Starship Child Health, Te Toka Tumai Auckland, Te Whatu Ora Health New Zealand, Auckland, New Zealand; 9 University of Otago, Christchurch, Otago, New Zealand; 10 Department of Pediatrics, Yale School of Medicine, Yale University, New Haven, CT, United States

**Keywords:** digital health, well-being app, type 1 diabetes, adolescents and young adults, diabetes distress, qualitative study

## Abstract

**Background:**

Individuals living with type 1 diabetes (T1D) are at an increased risk of experiencing psychological distress; however, there remains a scarcity of scalable and widely accessible support services, particularly for adolescents and young adults. To address this gap, digital mental health interventions are becoming an increasingly important area of innovation in diabetes care.

**Objective:**

This study aimed to explore qualitative feedback regarding the “Lift: Thriving with Diabetes” (*Lift*) well-being app, designed to support emotional well-being among adolescents and young adults with T1D, which was recently tested in a 12-week feasibility trial conducted in New Zealand and the United States.

**Methods:**

Of the 59 adolescents and young adults and 22 support people who participated in the main *Lift* feasibility trial, 13 agreed to participate in this secondary qualitative study. Participants attended a virtual focus group or 1-on-1 interview to discuss their experiences using the app and to explore their perspectives on the app’s engagement, functionality, and perceived impact on well-being and diabetes-related coping. Transcribed audio recordings were analyzed using directed content analysis, guided by the Mobile Application Rating Scale end-user framework (with topics of engagement, functionality, aesthetics, and information quality) and interpreted from a realist theoretical position.

**Results:**

In total, 9 adolescents and young adults (mean age 21.5, SD 2.06 years; n=5, 56% men) and 4 support people (2 fathers, 1 friend, and 1 partner; mean age 31.3, SD 18.92 years; n=2, 50% men) completed interviews. Overall, participants viewed *Lift* as engaging, easy to use, and emotionally impactful. The most positive feedback focused on the app’s interactive features, particularly a well-being tree that “grew” with increased engagement, and its “calming” visual aesthetics. Users also reported meaningful emotional or behavioral impact, particularly in promoting connection, self-awareness, and practical coping strategies in living with T1D. However, user feedback also highlighted areas for improvement, including the need for improved content pacing, personalization, connection with other digital health tools, and greater gamification to sustain long-term engagement. Participants consistently expressed a desire for content tailored to their age, role (eg, support person vs young person), and personal preferences (eg, voice, pace, tone, and interactivity).

**Conclusions:**

Findings underscore the potential of user-driven, emotionally intelligent digital tools to enhance well-being and connection for young people with T1D, as well as their support people. These insights can inform the refinement of *Lift* and the development of broader digital health interventions aimed at promoting well-being and fostering meaningful, sustained impact.

## Introduction

### Background

Individuals living with type 1 diabetes (T1D) are at increased risk for psychological distress [1,2], with notably higher rates of psychological and psychiatric disorders than those in the general population [3-5]. These rates are particularly pronounced among adolescents and young adults, whose unique developmental stage involves navigating tasks such as increasing autonomy and peer integration, which often conflict with the structured and demanding nature of diabetes management [6]. Such psychosocial challenges both disrupt and are exacerbated by the self-management requirements of T1D, creating a burdensome and often isolating cycle of distress [7,8].

Despite the high prevalence of psychosocial distress in this population, there remains a scarcity of targeted mental health services for adolescents and young adults with T1D. Existing supports are often fragmented, poorly integrated into routine care, or inaccessible due to geographical, financial, or systemic barriers [9,10]. Moreover, the emotional toll of T1D extends beyond those diagnosed; caregivers, siblings, and other loved ones often experience significant psychological strain, further impacting family functioning and the support environment for the young person [11-13]. This highlights a pressing need for interventions that address not only the clinical management of T1D but also the broader psychosocial challenges faced by adolescents and young adults and their families.

### Digital Health Interventions in Diabetes Care

Digital health interventions have been widely used as an avenue of health promotion and behavior change across a variety of health conditions [14,15] and are emerging as an increasing focus of innovation in diabetes care [16]. For diabetes, there exist a handful of commercially available apps, although these are largely designed to support the achievement of glycemic targets measured by hemoglobin A_1c_ (HbA_1c_) rather than the management of well-being more broadly [17]. Indeed, there is a gap in the digital health market for well-being apps specifically designed for individuals with diabetes.

Broadly, digital tools are well-suited to adolescents and young adults, who are often referred to as “digital natives” [18] due to their lifelong exposure to and comfort with technology. This familiarity provides a valuable opportunity to deliver engaging, scalable, and accessible interventions that align with the lifestyles and communication preferences of adolescents and young adults. In this context, digital platforms offer a compelling opportunity for delivering psychosocial support, facilitating education, and promoting self-management in a way that is both developmentally appropriate and responsive to the realities of living with T1D. In exploring methods for supporting self-management, well-being, and education, particularly among youth, digital tools are a growing solution.

In developing engaging and efficacious digital health interventions, the integration of end-user feedback is essential [19]. Such approaches allow a more granular understanding of what users want and what will keep them engaging with the interventions in a meaningful way. Maintaining user engagement, uptake, and adherence is a perennial challenge in digital health, especially among adolescents and young adults, who may quickly disengage from tools they perceive as irrelevant, unappealing, or burdensome [20].

In response to such challenges, increasing extant work has been conducted to assess digital health interventions. The Mobile Application Rating Scale seeks to assess the quality of mobile health interventions, while the uMARS (end-user version of the Mobile Application Rating Scale) framework is an end-user version of this tool [21]. By evaluating domains such as engagement, functionality, aesthetics, and information quality, the uMARS enables insight into which features are likely to support (or undermine) user interaction with an app. When used alongside co-design methods, tools such as uMARS can guide the iterative development of digital interventions, helping to ensure that they are both evidence-based and user-informed. Such approaches not only improve the likelihood of initial engagement but also enhance long-term adherence by fostering greater relevance, usability, and acceptability through direct collaboration with intended end users and ensuring that their lived experiences, needs, and preferences shape the intervention from the outset.

### Rationale for This Study

“Lift: Thriving with Diabetes” (*Lift*) is a digital well-being app designed to assist young people living with T1D and the people who support them (eg, parents, friends, and partners), which was recently tested for feasibility and usability in a 12-week pilot study with 59 adolescents and young adults and 22 support people (SPs) in New Zealand (NZ) and the United States [22]. It demonstrated promising findings in terms of safety, acceptability, and engagement, with retention and completion rates greater than 80%. The pilot study also demonstrated promising within-group improvements in psychological outcomes over time. The development of *Lift* was driven by a recognized unmet need for accessible psychosocial support for adolescents and young adults within the T1D community. Few digital interventions have been specifically designed to address the well-being of adolescents and young adults with T1D, and fewer still have been developed in collaboration with the young people and their support networks whom they are intended to serve.

This study aimed to qualitatively explore user feedback of *Lift*, focusing on both individual adolescent and young adult users and adolescent and young adult–SP dyads. Specifically, this work sought to understand users’ perspectives on the app’s engagement, functionality, and perceived impact on well-being and diabetes-related coping. By capturing rich, in-depth accounts of user experience, this study provides early insights into the acceptability and potential utility of a psychosocially oriented digital intervention for adolescents and young adults living with T1D, informing the refinement of *Lift* and similar well-being apps.

## Methods

### Participants

*Lift* was recently tested in a 12-week feasibility trial with 59 adolescents and young adults and 22 SPs in NZ and the United States [22]. In total, 13 participants (n=9, 15% adolescents and young adults and n=4, 18% SPs) from the feasibility trial agreed to participate in this secondary qualitative study.

### Intervention

“Lift: Thriving with Diabetes” is an adaptation of an existing well-being app [23] that was subsequently tailored for youth living with T1D [24]. *Lift* offers an evidence-based intervention consisting of seven core modules: (1) “Feel,” (2) “Relax,” (3) “Be Kind to Yourself,” (4) “Gratitude,” (5) “Connect,” (6) “Look After Your Body,” and (7) “Goal Setting.” Taken together, the modules aim to enhance users’ overall well-being and are developed based on strategies from cognitive behavioral therapy, mindful self-compassion, psychoeducation, positive psychology, and mindfulness. The original app has shown efficacy in improving psychological outcomes in adolescents and young adults without diabetes in a randomized controlled trial [23], and further information about the content and theory underpinning *Lift* is available in a protocol paper [25].

On the basis of feedback from 34 adolescents and young adults with T1D and health care professionals working in diabetes in NZ [24], the 7 modules of the original app were modified to be diabetes-specific, lived experience videos were included, and an SP module was developed. The gamification component was also enhanced, with the badges in the original app being replaced with a “well-being tree” that grows as modules and exercises are completed. *Lift* has both NZ and US versions, with culturally congruent imagery, phrases, and voice-overs for each country.

### Study Design

This qualitative study used 1-on-1 interviews and focus groups to evaluate user experiences of *Lift*, analyzed using directed content analysis. Recruitment occurred between July 2023 and December 2023. Both adolescent and young adult participants (aged 16 to 25 years) and SPs provided assent and/or informed consent and were recruited from sites within NZ and the United States in the context of their participation in a larger-scale feasibility trial of *Lift* [22]; participants were approached via email to ascertain interest in participating in this secondary qualitative study. In NZ, young people aged ≥16 years were able to provide their own informed consent. In the United States, participants aged <18 years provided assent, with informed consent obtained from a parent or caregiver.

### Focus Group and Individual Interview Facilitators

Participants could choose whether they wanted to participate in a focus group or an individual interview to accommodate scheduling needs. The focus groups in this study were cofacilitated by study authors (KMB [female] and ALB [female]), and 1-on-1 interviews were facilitated independently by ALB. At the time of data collection, both study authors had prior experience in facilitating group sessions with adolescents and conducting focus groups. Both KMB and ALB hold doctorate degrees in health psychology and are registered health psychologists. Neither KMB nor ALB had any prior relationship with the participants.

### Procedure

Both adolescents and young adults and caregivers who agreed to participate in this secondary study attended virtual focus groups or 1-on-1 interviews. A semistructured interview schedule (Table S2 in Multimedia Appendix 1), based on the uMARS framework [21], was developed to guide participants in discussing their experience of using the app; however, participants could discuss other diabetes-related topics outside the uMARS framework if desired. Focus groups and 1-on-1 interviews were conducted online using Zoom (Zoom Communications Inc) video conferencing; both audio and video were recorded. Transcribed audio recordings of the interviews and focus groups were analyzed using directed content analysis. Recruitment for this study was closed after data saturation was reached.

### Data Analysis

Given that predefined open-ended questions from the uMARS framework were used to explore engagement with the app, directed content analysis, a qualitative method guided by existing frameworks or theories [26], was used, along with a realist theoretical position [27]. NVivo (version 15; Lumivero LLC) was used to facilitate content analysis of qualitative data, and the coding tree was housed in Microsoft Excel. Analysis was abductive, with predetermined deductive codes from the uMARS framework and inductive codes flexibly added during the coding process as needed, which is in alignment with directed content analysis methods and recommended when exploring user experience with digital health interventions [28]. Initial deductive codes were agreed upon by the research team, and 2 researchers (KB and KHG) coded the data. Following initial deductive coding, the 2 coders completed additional inductive coding and then met with the core research team to agree on final categories. Any coding disagreements were resolved via consensus between the coders, and categories were finalized through discussion until agreement was reached within the research team.

### Ethical Considerations

The Consolidated Criteria for Reporting Qualitative Research (COREQ) checklist (Checklist 1) was followed to report the qualitative study findings. All research methods were approved by the Health and Disability Ethics Committees in NZ (2023 EXP 13982) and the Yale Institutional Review Board (2000034956) prior to study advertisement and recruitment. Informed written consent or assent was provided by participants in advance of their participation in this study. All data were deidentified. Individuals were provided with a NZ $50 (US $28) gift voucher to compensate them for their participation.

## Results

### Participant Characteristics

A total of 13 participants were enrolled in this study. Of the 81 participants (n=59, 73% adolescents and young adults and n=22, 27% SPs) initially approached from the *Lift* feasibility study [22], 16 (20%) indicated interest in participating in focus groups or interviews. Subsequently, 3 (19%) participants did not respond to prompts for scheduling. Data saturation was reached after approximately 12 (92%) participants had participated in focus groups or interviews; therefore, no additional potential participants were contacted. Demographic information for the sample, stratified by adolescent and young adult (n=9, 69%) and SPs (n=4, 31%), can be found in Table 1.

**Table 1.** Demographic characteristics of the participants.

Variables	AYA^a^ participants (n=9)	Support people (n=4)
Age (years), mean (SD)	21.50 (2.06)	35.25 (18.92)
Gender, n (%)		
Man	5 (55.56)	2 (50.00)
Woman	3 (33.33)	2 (50.00)
Nonbinary	1 (11.11)	0 (0)
Ethnicity, n (%)		
Chinese	1 (11.11)	0 (0)
Indian	1 (11.11)	0 (0)
Māori^b^	1 (11.11)	1 (25.00)
New Zealand European	2 (22.22)	3 (75.00)
White American	4 (44.44)	0 (0)
Relation to AYA, n (%)		
Father	N/A^c^	2 (50.00)
Partner	N/A	1 (25.00)
Friend	N/A	1 (25.00)

a AYA: adolescent and young adult.

b Indigenous people of New Zealand.

c N/A: not applicable.

Two 1-on-1 interviews were conducted with adolescents and young adults who could not attend the focus groups. Three focus groups were conducted: one with adolescents and young adults (n=3, 33%), one with SPs (n=3, 75%), and one combined group that included both adolescents and young adults and SPs (n=5, 38%). On average, the focus groups were 64 (SD 8.59) minutes in duration and 1-on-1 interviews were 44 (SD 1.53) minutes in duration.

### User Perspectives: Engagement

The “Engagement” domain of the uMARS framework was operationalized as factors relating to entertainment, interest, customization, and interactivity and was divided into 2 subcategories: “more engaging” and “less engaging.” Broadly, “Engagement” was the largest source of data from participants and was a positively skewed domain. Many participants (n=10, 77%) reported feeling positively engaged, citing features such as the well-being tree, interactive modules, journaling, and check-ins as motivating and rewarding:

I quite enjoyed like the check-ins...you had to take a minute to stop and reflect, and actually...recognise how you were feeling.

[SP (father), 52 years, NZ]

The visual growth of the tree, in particular, was repeatedly described as an effective incentive to return to the app. Interactive elements, such as prompts following videos, repeatable activities, and opportunities for self-reflection, were appreciated for fostering personal accountability and emotional engagement:

I liked how interactive it was and how you could repeat activities. It meant to me that if I wanted the comfort of doing it over, I could easily do that.

[Adolescent and young adult, 18 years, United States]

However, many participants (n=8, 62%) expressed declining engagement over time, particularly after completing all available modules:

It got boring rather quickly. Once all the modules are done and you figure out all the ones that work for you personally, there isn’t...much else the app can really provide from that point onwards. Plus, once the tree reaches its max height, there is even less incentive to go onto the app.

[Adolescent and young adult, 17 years, NZ]

The pace of content delivery was also a barrier to engagement; several participants indicated that they wanted to speed up the delivery of content (n=6, 46%). Although some appreciated the reflective function of check-ins, others found the repetition disengaging:

Having to check in every night became annoying.

[Adolescent and young adult, 22 years, United States]

Some users felt that the tone of the app, especially the narration and emotional identification tools, was overly simplistic or better suited to a younger audience (n=4, 31%). Only 2 (15%) participants noted a preference for alternative content formats (eg, written text instead of video) and requested features such as subtitles. A common thread among disengaged users was a lack of novelty and progression. Despite these criticisms, more participants reported feeling more engaged than disengaged overall.

### User Perspectives: Functionality

Functionality, defined as performance, ease of use, navigation, and gestural design, was generally rated positively, with some participants (n=5, 38%) describing the app as easy to use and intuitively designed. The setup process was straightforward for nearly all users:

I didn’t have too much trouble with it with the downloading and setting it up, it was relatively user-friendly.

[SP (father), 54 years, NZ]

Participants appreciated the dashboard layout, the ability to track progress, and the flexible structure that allowed them to complete modules in their preferred order:

It’s easy to navigate, so you know what to do, what you’ve done, what’s coming up. I liked that aspect of it.

[Adolescent and young adult, 22 years, United States]

However, a subset of participants reported issues with navigation (n=2, 15%) and technical performance (n=2, 15%):

I found it confusing to know what modules I needed to do when*.*

[Adolescent and young adult, 17 years, NZ]

In total, 2 (15%) users experienced app crashes or problems with audio playback. Lack of accessibility features, such as video subtitles or text alternatives, was another concern:

I wish it was transcribed...I didn’t want everyone else to know what I’m doing.

[SP (father), 51 years, NZ]

I prefer to read rather than listen.

[Adolescent and young adult, 22 years, Unites States]

Overall, the app’s functionality was viewed as a strength, particularly in terms of layout, usability, and user autonomy, although minor navigation difficulties and accessibility limitations were noted by some participants.

### User Perspectives: Aesthetics

In terms of aesthetics (factors relating to layout, graphics, and visual appeal), data were divided into 2 subcategories: “positive” and “negative.” Many participants (n=7, 54%) expressed positive views on the app’s design, particularly its visual appeal and layout. The use of green was frequently highlighted (n=5, 38%) as contributing to a peaceful and comforting aesthetic:

I think the app has quite a gentle element of design to it and I think it was quite peaceful.

[Adolescent and young adult, 20 years, NZ]

It’s...made to look very friendly and assuring.

[Adolescent and young adult, 19 years, NZ]

Some users also reflected that they liked the voices used within the app (n=4, 31%). Some NZ participants noted, in particular, that they valued the personalization of the app using commonplace language and voice actors with NZ accents:

I liked that they seemed to be Kiwi...It definitely seemed to be...in touch with New Zealand.

[Adolescent and young adult, 19 years, NZ]

I liked how they incorporated the use of te reo [indigenous language of New Zealand].

[Adolescent and young adult, 16 years, NZ]

However, some participants found these components to be negative; several participants noted that they did not find the colors appealing (n=3, 23%). Additionally, although many enjoyed the tone of the narration, others found the voices overly rehearsed (n=5, 38%):

Me personally I don’t like the...“calming voice.” It’s a personal preference of mine I don’t like people talking to me in that certain tone.

[Adolescent and young adult, 20 years, United States]

A small number of participants (n=2, 15%) also suggested that the visuals could more directly reflect diabetes-related themes to enhance the app’s relevance to its target audience.

### User Perspectives: Information

The “Information” domain of the uMARS framework, defined by content quality, clarity, and perceived relevance, received largely positive feedback. Several participants appreciated the depth and range of the modules, with specific praise for “Be Kind and Relax,” which was the most frequently mentioned (n=6, 46%) as helpful.

Other modules, including “Be Thankful,” “Look After Your Body,” and those incorporating breathing and journaling exercises, were also described as useful, especially when they felt practical or easily incorporated into daily routines. However, some feedback suggested that the app’s content was not always relevant for SPs (n=2, 15%):

A lot of the things are targeted at a person with diabetes, and I’m the support person*.*

[SP (father), 51 years, NZ]

Several participants (n=3, 23%) expressed personal preferences or limitations that influenced how helpful they found certain modules—for example, difficulty engaging with meditation due to chronic pain or discomfort with emotional content:

Breathing or meditation, like that’s not for me.

[Adolescent and young adult, 22 years, United States]

Certain modules are kind of fit for different personality types.

[Adolescent and young adult, 20 years, United States]

Two felt that the tone or framing of certain concepts could be off-putting:

Be Thankful...felt a little suggestive of “hey, it could be worse*.”*

[Adolescent and young adult, 21 years, United States]

### User Perspectives: Recommendations for Future Development

Participants shared a range of suggestions for how the app could be enhanced in future iterations. A central area of focus was the well-being tree, which most participants (n=9, 69%) valued but wished could evolve further to include customizable or seasonal elements, new objects (eg, flowers or gardens), and even motivational consequences such as wilting:

I can’t let my digital tree die, yeah. So, I think that would be a good motivator.

[Adolescent and young adult, 22 years, NZ]

Some participants (n=4, 31%) also proposed alternatives to the tree, including growing spaceships or gardens, to support greater personal relevance and customizability.

Some participants (n=3, 23%) also highlighted opportunities to integrate the app with existing digital tools and networks:

It would be really cool to see this kind of app and the diabetes tracking combined with one of the health watches...to see how my blood sugar reacts to specific things.

[Adolescent and young adult, 24 years, United States]

if you want to make it New Zealand specific you could always integrate it with a lot of the community groups we have like Diabetes Youth Auckland.

[Adolescent and young adult, 20 years, NZ]

Personalization was another interest. Overall, 2 (15%) users requested the ability to tailor the app to their age, stage of life, and content preferences:

I would make it have settings for specific age groups/stages of life for a more personable experience.

[Adolescent and young adult, 21 years, United States]

I would give different options for voices and tones.

[Adolescent and young adult, 20 years, United States]

SPs (n=3, 23%) called for more targeted content and pathways:

Maybe there could be like a category around like parents or friends or something.

[SP (friend), 16 years, NZ]

Some participants (n=3, 23%) also saw potential in incorporating more lived experience content to foster connection and normalize challenges:

The lived experiences part is cool...maybe a moderated submission element where people could even anonymously post lived experiences would be great.

[Adolescent and young adult, 20 years, NZ]

Others envisioned video or podcast libraries featuring community members and health professionals. Finally, some participants (n=5, 38%) wanted more interactivity to maintain engagement:

More activities in between the words could be more engaging*.*

[Adolescent and young adult, 21 years, United States]

you could almost have...friend quests...like seeing each other’s trees*.*

[SP (father), 51 years, NZ]

### User Perspectives: Impact of the Lift App

Many participants (n=7, 54%) described the app as having a meaningful emotional or behavioral impact, particularly in promoting connection, self-awareness, and practical coping strategies. For 1 (8%) adolescent and young adult and SP dyad, the shared experience of using the app strengthened their sense of support:

It was a really fun experience to be able to actually do something together...kind of like Duo Lingo...stuff you can do daily.

[SP (father), 54 years, NZ]

Others appreciated the app’s emphasis on emotional reflection and noted the capacity for building longer-term skills:

It made me more self-aware of my emotions and inspired me to control what I can to change those feelings.

[SP (partner), 22 years, NZ]

I liked how it allowed me to build better strategies to cope with having diabetes.

[Adolescent and young adult, 20 years, NZ]

One participant also highlighted a sense of common humanity derived from the app:

It was nice to hear from other people with diabetes because it makes you feel less alone*.*

[Adolescent and young adult, 16 years, NZ]

## Discussion

### Principal Findings

This qualitative study builds on a recently published feasibility evaluation of the *Lift* digital health intervention for adolescents and young adults with T1D, which demonstrated promising preliminary findings and showed that *Lift* is safe, acceptable, and engaging among users [22]. This study aimed to capture user experiences, perceived impact, and suggestions for improving future iterations of the app. Overall, participants in this study described *Lift* as engaging, easy to use, and impactful and made suggestions for future improvements, including personalization and greater gamification.

Participants consistently expressed a desire for content tailored to their age, role (eg, SP vs young person), and personal preferences (eg, voice, pace, tone, and interactivity). This aligns with existing research showing that personalization can enhance engagement with digital health interventions by increasing the perceived relevance of content, thereby improving user motivation [29]. However, evidence also cautions that misaligned content, when not well matched to user needs or preferences, can lead to disengagement [30]. Indeed, this was echoed by some of the parent participants, who felt that they were not the “target market” for certain content, particularly content specific to diabetes.

For self-guided digital health interventions such as *Lift*, fostering engagement requires balancing broad appeal with delivering evidence-based, specific, and useful content. Moreover, it is difficult to predict the specific preferences and characteristics of end users, suggesting that future iterations of *Lift* and similar digital interventions could benefit from modular and adaptive content pathways that allow users to autonomously tailor their experience (such as content targeted to parents of adolescents and young adults living with diabetes). Indeed, this aligns with the self-determination theory, which, in digital health contexts, postulates that autonomy and perceived relevance are essential for sustaining intrinsic motivation to engage [31].

Qualitative data collected in this study highlighted that participants appreciated shared experiences (eg, stories, lived experience videos, and quotes), and many proposed interactive or social features (eg, forums, friend quests, and community groups) as ways to further improve the app. Participants highlighted that this content reduced feelings of isolation and enhanced the impact of *Lift*. These insights can be contextualized within some of the core frameworks underpinning the app, including self-compassion and acceptance and commitment therapy, which emphasize the role of common humanity and connectedness as buffers against shame and stress, particularly in diabetes [32]. This is a particularly impactful finding when taking into consideration the high levels of self-stigma and shame in diabetes [33].

Research supports social connection as a predictor of positive coping and a powerful mediator of diabetes distress [34,35], and previous research highlights that youth with diabetes want peer support features in digital interventions [36]. However, what remains unclear in the context of digital health is how to sustainably establish safe and moderated online spaces, particularly for users who are minors. At least initially, further work could be done to establish paired interactive features (such as “friend quests” and challenges), and future development could explore how platforms for community contribution or interaction could safely and sustainably be established, thus further supporting user retention, engagement, and the provision of connection and common humanity.

In keeping with findings regarding the role of social features in incentivizing engagement, interactive features were also an area of rich data in this study. Participants noted that app features such as the well-being tree, check-ins, and progress indicators were viewed as motivating; however, these needed more variety and novelty to sustain engagement. Many participants viewed these features as not just fun but also as motivational tools that helped them re-engage with the app and their self-care routines.

These findings can be contextualized within behavioral design theory, particularly through the value of design components such as feedback loops and progress indicators, which can increase engagement by satisfying the psychological need for competence and achievement [37]. For instance, the idea of the tree wilting when not used (and flourishing when attended to) mirrors habit loop mechanics, in which behavior is reinforced by visible progress and mild consequences for inaction. This is consistent with other digital health research, which has found that gamification increases engagement and facilitates greater behavior change [38,39]. It stands to reason that increasing gamification within *Lift* may increase and sustain both motivation and engagement through the provision of small, meaningful rewards.

There are also limitations to this study. It was a small qualitative study with a relatively homogeneous group of participants; therefore, generalized conclusions cannot be made. The participants who volunteered for this study after completing the *Lift* feasibility study may also represent more engaged users. Finally, this study did not explore cultural and health care system differences between the 2 study sites (NZ and United States), which could have affected the overall findings.

### Conclusions

This qualitative study highlights user experiences and key benefits of *Lift* from the perspective of adolescents and young adults living with diabetes and their SPs. Participants reported improved emotional awareness, stress reduction, and reflective practices attributed to the app. These findings reinforce the value of emotionally intelligent, user-driven design in digital health. As adolescents and young adults and their support networks continue to navigate the complexities of living with diabetes, digital tools such as *Lift* show promise not just in promoting self-care, but also in fostering connection, agency, and a sense of shared humanity. This study also offers key insights both for future iterations of the *Lift* app and for digital health interventions more broadly. Future research should explore how such tools can be scaled, personalized, and embedded into everyday care, turning small digital moments into meaningful, sustained impact.

## Supplementary material

10.2196/79896 Multimedia Appendix 1 Table for the interview schedule for adolescents and young adults and their caregivers.

10.2196/79896 Checklist 1 COREQ Checklist

## Abbreviations

COREQ: Consolidated Criteria for Reporting Qualitative Research
Lift: Lift: Thriving with Diabetes
NZ: New Zealand
SP: support person
T1D: type 1 diabetes
uMARS: end-user version of the Mobile Application Rating Scale

## References

[1] Morrissey EC, Casey B, Dinneen SF, Lowry M, Byrne M (2020). Diabetes distress in adolescents and young adults living with type 1 diabetes. Can J Diabetes.

[2] Stahl-Pehe A, Glaubitz L, Bächle C (2019). Diabetes distress in young adults with early-onset type 1 diabetes and its prospective relationship with HbA_1c_ and health status. Diabet Med.

[3] Collins MM, Corcoran P, Perry IJ (2009). Anxiety and depression symptoms in patients with diabetes. Diabet Med.

[4] Hanson CL, De Guire MJ, Schinkel AM, Kolterman OG, Goodman JP, Buckingham BA (1996). Self-care behaviors in insulin-dependent diabetes: evaluative tools and their associations with glycemic control. J Pediatr Psychol.

[5] Kakleas K, Kandyla B, Karayianni C, Karavanaki K (2009). Psychosocial problems in adolescents with type 1 diabetes mellitus. Diabetes Metab.

[6] Merikangas KR, He JP, Burstein M (2010). Lifetime prevalence of mental disorders in U.S. adolescents: results from the National Comorbidity Survey Replication--Adolescent Supplement (NCS-A). J Am Acad Child Adolesc Psychiatry.

[7] McCoy RG, Kidney RS, Holznagel D, Peters T, Madzura V (2019). Challenges for younger adults with diabetes. Minn Med.

[8] Pyatak EA, Sequeira PA, Whittemore R, Vigen CP, Peters AL, Weigensberg MJ (2014). Challenges contributing to disrupted transition from paediatric to adult diabetes care in young adults with type 1 diabetes. Diabet Med.

[9] Shulman R, Luo J, Shah BR (2018). Mental health visits and low socio-economic status in adolescence are associated with complications of type 1 diabetes in early adulthood: a population-based cohort study. Diabet Med.

[10] Nakhla M, Shulman R, Dimeglio L (2021). Mental health matters: limited support remains a barrier to optimal care for youth with diabetes. Can J Diabetes.

[11] Cao VT, Anderson BJ, Eshtehardi SS (2021). “We are a family with diabetes”: parent perspectives on siblings of youth with type 1 diabetes. Fam Syst Health.

[12] Chan KK, Shorey S (2022). Experiences and needs of children with siblings diagnosed with type 1 diabetes: a mixed studies systematic review. J Pediatr Nurs.

[13] Whittemore R, Jaser S, Chao A, Jang M, Grey M (2012). Psychological experience of parents of children with type 1 diabetes: a systematic mixed-studies review. Diabetes Educ.

[14] Philippe TJ, Sikder N, Jackson A (2022). Digital health interventions for delivery of mental health care: systematic and comprehensive meta-review. JMIR Ment Health.

[15] Bernstein CM, Stockwell MS, Gallagher MP, Rosenthal SL, Soren K (2013). Mental health issues in adolescents and young adults with type 1 diabetes: prevalence and impact on glycemic control. Clin Pediatr (Phila).

[16] Garner K, Boggiss A, Jefferies C, Serlachius A (2022). Digital health interventions for improving mental health outcomes and wellbeing for youth with type 1 diabetes: a systematic review. Pediatr Diabetes.

[17] Maharaj A, Lim D, Murphy R, Serlachius A (2021). Comparing two commercially available diabetes apps to explore challenges in user engagement: randomized controlled feasibility study. JMIR Form Res.

[18] Butler S, Sculley D, Santos DS (2020). Usability of eHealth and mobile health interventions by young people living with juvenile idiopathic arthritis: systematic review. JMIR Pediatr Parent.

[19] Bevan Jones R, Stallard P, Agha SS (2020). Practitioner review: co-design of digital mental health technologies with children and young people. J Child Psychol Psychiatry.

[20] Malloy J, Partridge SR, Kemper JA, Braakhuis A, Roy R (2023). Co-design of digital health interventions with young people: a scoping review. Digit Health.

[21] Stoyanov SR, Hides L, Kavanagh DJ, Wilson H (2016). Development and validation of the user version of the Mobile Application Rating Scale (uMARS). JMIR Mhealth Uhealth.

[22] Serlachius A, McClintock J, Boggiss A (2025). Lifting the wellbeing of adolescents and young adults with type 1 diabetes: a feasibility study of the LIFT app. Int J Med Inform.

[23] Thabrew H, Boggiss AL, Lim D (2022). Well-being app to support young people during the COVID-19 pandemic: randomised controlled trial. BMJ Open.

[24] Garner K, Thabrew H, Lim D, Hofman P, Jefferies C, Serlachius A (2023). Exploring the usability and acceptability of a well-being app for adolescents living with type 1 diabetes: qualitative study. JMIR Pediatr Parent.

[25] Serlachius A, Schache K, Boggiss A (2020). Coping skills mobile app to support the emotional well-being of young people during the COVID-19 pandemic: protocol for a mixed methods study. JMIR Res Protoc.

[26] Hsieh HF, Shannon SE (2005). Three approaches to qualitative content analysis. Qual Health Res.

[27] Couch D, Okoko JM, Tunison S, Walker KD (2023). Varieties of Qualitative Research Methods: Selected Contextual Perspectives.

[28] Harrison Ginsberg K, Babbott K, Serlachius A (2024). Exploring participants’ experiences of digital health interventions with qualitative methods: guidance for researchers. J Med Internet Res.

[29] Morrison LG (2015). Theory-based strategies for enhancing the impact and usage of digital health behaviour change interventions: a review. Digit Health.

[30] Updegraff JA, Sherman DK, Luyster FS, Mann TL (2007). The effects of message quality and congruency on perceptions of tailored health communications. J Exp Soc Psychol.

[31] Şener B, Umulu S, Yilmaz AO, Bohemia E, Buck L, Grierson H (2022). Proceedings of the 24th International Conference on Engineering and Product Design Education (E&PDE 2022).

[32] Onu DU, Obi-keguna CN, Oguguam ON, Ajaero CK, Igwe EJ (2025). Social support may buffer, to an extent, the impact of stigma on health-related quality of life among type 2 diabetes mellitus patients. Discov Public Health.

[33] Speight J, Holmes-Truscott E, Garza M (2024). Bringing an end to diabetes stigma and discrimination: an international consensus statement on evidence and recommendations. Lancet Diabetes Endocrinol.

[34] Chan CK, Cockshaw W, Smith K, Holmes-Truscott E, Pouwer F, Speight J (2020). Social support and self-care outcomes in adults with diabetes: the mediating effects of self-efficacy and diabetes distress. Results of the second diabetes MILES - Australia (MILES-2) study. Diabetes Res Clin Pract.

[35] Luo D, Cai X, Wang H, Wang Y, Xu J (2024). The role of peer social relationships in psychological distress and quality of life among adolescents with type 1 diabetes mellitus: a longitudinal study. BMC Psychiatry.

[36] Boggiss AL, Consedine NS, Schache KR (2021). Exploring the views of adolescents with type 1 diabetes on digital mental health interventions: what functionality and content do they want?. Diabet Med.

[37] Fogg B (2009). Persuasive ’09: Proceedings of the 4th International Conference on Persuasive Technology.

[38] Berglund A, Jaarsma T, Berglund E, Strömberg A, Klompstra L (2022). Understanding and assessing gamification in digital healthcare interventions for patients with cardiovascular disease. Eur J Cardiovasc Nurs.

[39] King D, Greaves F, Exeter C, Darzi A (2013). “Gamification”: influencing health behaviours with games. J R Soc Med.

