# Supplementary material for: Exploring User Experiences With the Lift App for Emotional Well-Being Among Youth With Type 1 Diabetes: Qualitative Study
Source: JMIR Diabetes. 2026 May 28;11:e79896. doi: 10.2196/79896 (PMC13218564; doi:10.2196/79896)
Supplement: Multimedia Appendix 1 [file diabetes-v11-e79896-s001.pdf]

Supplementary Table 2. Interview Schedule for AYA and Caregivers

Interview Schedule
**Setting the Scene** *(AB)*<br>• Greetings and introduction of moderators, icebreaker activity<br>• Ground rules, thank participants for coming, confidentiality<br>o Aim of focus group = opinions on content, usability, overall utility, and design of the app
**First impressions** *(KB)*<br>• Why did you choose to participate in the study? [a]<br>• What were your first impressions of the app?<br>• What did you like most? What made using the app a positive experience?<br>• What did you think could be better? What made the app difficult to use?
**Design** *(AB)*<br>• How would you describe the feel of the app?<br>• How did you feel about the colours and icons?<br>• What did you think of Ben and Ana?<br>• How did it feel to have a voice of someone with diabetes and the other of a support person? [b]
**Engagement/ features** *(AB)*<br>• What did you think of the well-being tree?<br>• What did you think about the daily well-being check-in?<br>o What options in the check-in future would you want to see in the future?<br>• How often did you re-visit an exercise? Did you use the "favourite feature"?<br>• Did anyone watch any of the lived experience videos? How did you find them?<br>o Some people suggested games. What type of games would you want to see?<br>• What other features would you like to see in the future?<br>• Speaking more broadly in terms of a program designed specifically for support people, what features/ functions would you like to see in the future? [a]<br>• When would be the most appropriate time to be offered an app or program? (i.e., at time of diagnoses, shortly after, anytime is an appropriate time) [a]<br>• For support people earlier on in their experience supporting a young adult with diabetes, what would be helpful for them in an app or program? [a]<br>• If a healthcare professional recommended an app or program to support you as a support person for a young adult with diabetes, what would make you more likely to use it? [a]<br>o What is the most feasible/ desirable length? How did you find 7 days? What would you think of something briefer versus more ongoing?

Support person *(KB)*

- Did you have a support person who was also using the LIFT app? [b]
  - If not, could you tell us why you chose not to have a support person? [b]
  - What was it like using the same app as your young adult/support person?
  - Did you notice any changes in them?
  - How did you talk about the app or it's included exercises with them?
  - Did you think it was useful that you were both using the app?

Modules *(AB)*

- What did you think of the modules? Did you find the information helpful?
- Which module was the most interesting/ helpful to you? Why?
- Which module did you dislike the most? Why?
- If we added more modules in the future, what would you want to see?
- What did you think of the content/ exercises in this module?
- Do you experience any difficulties completing any of the exercises in this module?
- What do you think could be improved in this module?

Wrap-up *(AB)*

- Would you recommend this app to your whānau/ friends with diabetes?
- How would you describe this app to someone you know?
- If we did one thing to improve the app, what would it be?
- Is there anything else you would like to tell us about your experience using LIFT?

'[a]' denotes questions only asked of support people; '[b]', questions only asked of AYA.
